# Layered habitats: An evolutionary model for present-day recreational needs

**DOI:** 10.3389/fpsyg.2022.914294

**Published:** 2022-11-30

**Authors:** Jonathan Stoltz

**Affiliations:** Department of People and Society, Swedish University of Agricultural Sciences, Alnarp, Sweden

**Keywords:** human habitats, landscape preferences, evolutionary aesthetics, cultural ecosystem services, outdoor recreation, health and wellbeing, restoration, perceived sensory dimensions

## Abstract

Urbanisation and lifestyle-related illnesses increase globally. This highlights the need to shape modern human habitats to support basic recreational needs, promoting such things as physical activity and restoration of high stress levels and cognitive fatigue. Previous research suggests eight perceived qualities in the outdoor environment, described as eight perceived sensory dimensions, as universally meaningful to people in this regard. However quite extensively studied in relation to various health and wellbeing outcomes, human sensitivity and appreciation for these qualities has not yet been explicitly analysed from an evolutionary perspective. This paper investigates their possible evolutionary roots and suggests an order for their development. This is linked with empirical findings on their relative capacity to support restoration of stress and cognitive fatigue. Qualities of earlier origin are suggested to correspond to older, more fundamental adaptations. Each subsequently developed quality implies an increased complexity of our environmental relations, associated with higher demands on more recently developed capacities. The proposed model thus links the more restorative Serene, Sheltered, Natural, and Cohesive perceived sensory dimensions with earlier stages of our development while the more demanding Diverse, Open, Cultural, and Social qualities are associated with more recent transitions. It might be of relevance when shaping modern human habitats from a health-promoting perspective, and have applications in the planning and design of, e.g., health care settings, rehabilitation gardens, urban green areas, recreational forests or other similar outdoor environments.

## Introduction

While urbanisation continues to increase (UN, United Nations, Department of Economic and Social Affairs, Population Division, 2019), noncommunicable, lifestyle-dependent and often stress-related, illness dominate globally (WHO, 2021). This highlights the necessity to shape modern human habitats to support people’s recreational needs; to promote such things as social cohesion, physical activity, and restoration from stress and attention fatigue. Here, perceived qualities in the outdoor environment play an important role. Several studies suggest eight perceived qualities, interpreted as eight perceived sensory dimensions, or PSDs (Grahn and Stigsdotter, 2010), as universally important to support complementary recreational needs: a Natural, a Cultural, a Cohesive, a Diverse, a Sheltered, an Open, a Cultural, and a Social quality, respectively (following Stoltz and Grahn, 2021). Not the least, they have been shown to be important in relation to restoration from stress and cognitive fatigue. Although quite extensively studied and employed in various practical and scientific contexts, these qualities have not yet been analysed along an evolutionary timeline, which potentially could explain their relative restorative influence and perhaps further support their proposed cross-cultural relevance (*ibid.*).

This paper has two main aims, (1) to show how the eight PSDs can be linked to different phases during human evolution, and (2) relate this to how the PSDs can support different levels of restoration. In addition, some suggestions are made for how the proposed model could be applied in the planning and design of modern human habitats, green areas, care settings, forests, etc., to support complementary recreational needs. The basic hypothesis developed is that the eight PSDs reflect a sum of adaptations made during several subsequent transitional phases of our evolution and development as a species. Over time, adaptations to novel environmental conditions have been integrated with older, more basic capacities, thus shaping modern perceptual biases and needs. Past environmental change and our ancestors’ subsequent adaptations are thus hierarchically represented in our psychophysiology in a layered, yet integrated way. This is not the least evident in the layered yet integrated structure of the human nervous system and brain, with more ancient, basic functions relatively preserved towards the core and more recent developments integrated on top of those (Aboitiz and Montiel, 2012; Wang, 2012; Hofman, 2014). With each developmental stage implying an increased complexity of our organism and of our environmental relations, this affects how the PSDs can support restoration of stress levels and cognitive fatigue.

## The perceived sensory dimensions model

**Figure 1:**
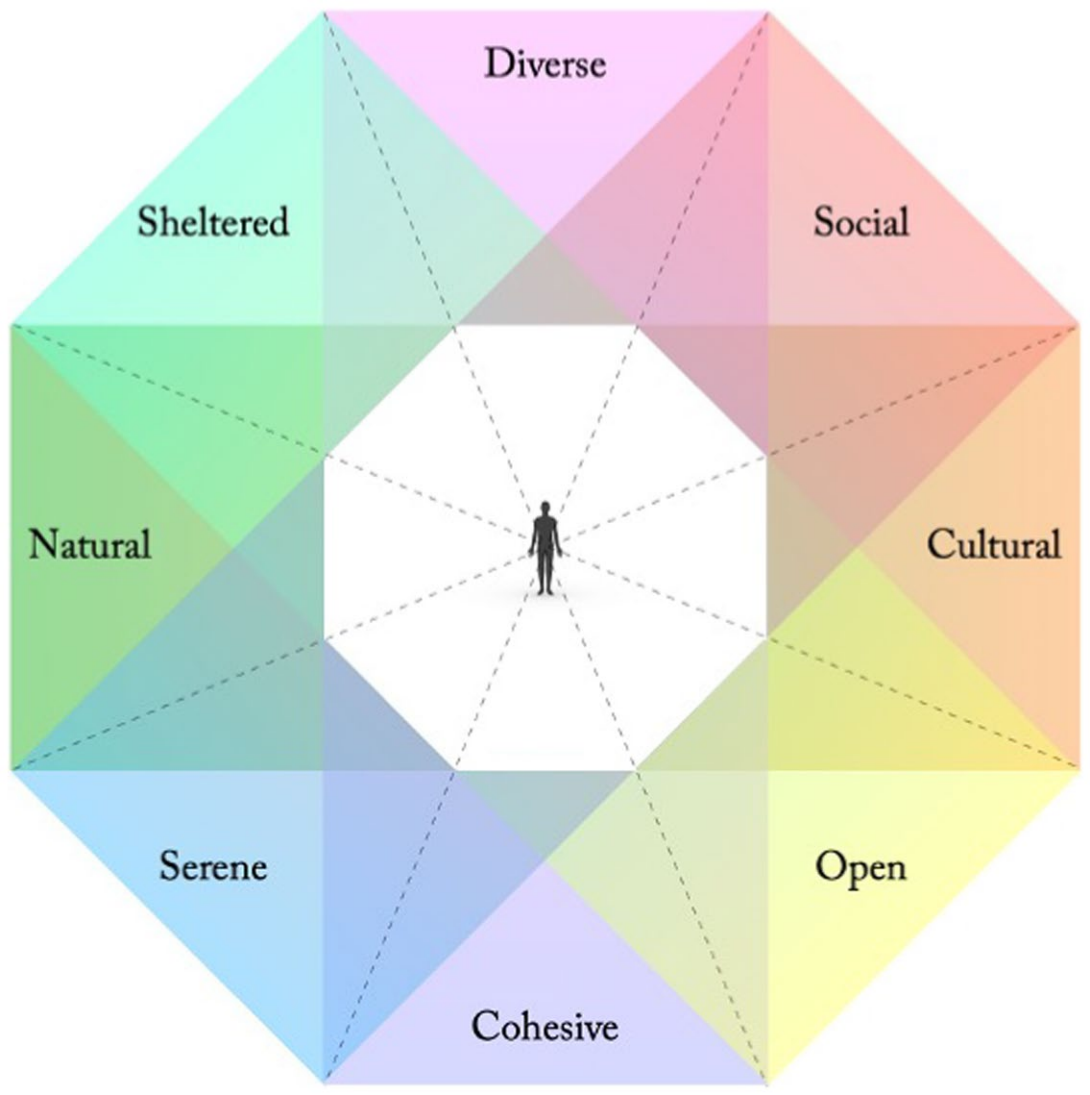
Eight PSDs along four axes of opposing qualities (Stoltz and Grahn, 2021). The closer together in the model, the more shared associations between qualities. Adjacent qualities thus often reinforce each other, while opposing qualities might weaken or contradict each other.

Several studies regarding needs and preferences in relation to recreational outdoor environments suggest that people are particularly sensitive to the availability of eight perceived qualities, or perceived sensory dimensions (PSDs; Grahn and Stigsdotter, 2010), to support their wellbeing (*ibid.*; Grahn, 1991; Grahn et al., 2005; Adevi and Grahn, 2012). They are defined from a human-centred perspective as classes of affordances (Gibson, 1979; Chemero, 2009); utilisable possibilities for meaningful behaviours and experiences in relation to current needs. As design principles the PSDs have been compared to basic “colours” in the environment, that can be supported to different degrees and mixed in various ways, shaped by all our sense modalities as well as our cognitive and physiological capacities (Stoltz, 2019; Stoltz and Grahn, 2021). As such, they have been interpreted along four axes of opposing qualities: A Natural – Cultural, a Cohesive – Diverse, a Sheltered – Open, and a Serene – Social axis (*ibid.*; Figure 1; Table 1).

**Table 1:** Eight perceived sensory dimensions, understood as complementary pairs of opposing qualities (following Stoltz and Grahn, 2021).

PSD	The environment affords
Natural	A Natural quality; a sense of the natural world, its distinctive shapes and patterns, its inherent force and power. The wild and untouched; free-growing vegetation, veteran trees, self-sown plants, and the passage of time without human intervention.
Cultural	A Cultural quality: a sense of the cultivated, crafted, and man-made, as opposed to the “self-made” or naturally developed. Distinct traces of human creative effort, culture, and history. Efforts of both past and present generations.
Cohesive	A Cohesive quality; a sense of spatial and structural cohesion and unity. A spacious, uninterrupted whole, a “world in itself” surrounding the visitor, possible to wander around within and explore.
Diverse	A Diverse quality; a sense of structural and ecological diversity. A large variety of different species of plants and animals. A multi-layered and structurally diverse vegetation, mixed with, e.g., water features.
Sheltered	A Sheltered quality; a sense of shelter, safety and protection. Often associated with smaller, relatively enclosed spaces, where sufficient openings and transparency still maintain good visibility and a connection with the outside world.
Open	An Open quality; a sense of openness and freedom from physical obstacles. Large open spaces with plenty of room to roam freely, to see far into the distance. Generous prospects and vistas, with unbroken sightlines.
Serene	A Serene quality; a sense of tranquillity, peace, serenity. Freedom from noise and disturbances. Peaceful sounds of nature. Absence of other people, signs, signals, or otherwise threatening or intrusive stimuli.
Social	A Social quality; a sense of bustling activity, people, and movement. A dense, lively place, with lots of social activities and interactions. Often particularly strong in urban settings, e.g., around cafés, shopping streets, squares, etc.

Versions of the PSDs has been investigated in more than 60 studies worldwide and results suggest their relevance across cultural contexts (Stoltz and Grahn, 2021). They have been analysed and evaluated in both urban and rural settings (e.g., de Jong et al., 2012; Stoltz and Schaffer, 2018), in rehabilitation gardens (Grahn et al., 2010; Pálsdóttir et al., 2018), and in forests environments (Stoltz et al., 2016; Stigsdotter et al., 2017). The impact on public health and wellbeing outcomes from the number of PSDs supported in people’s close-by living environment has been investigated in epidemiological studies. Björk et al. (2008) found an association with more PSDs perceived as supported in proximity to the dwelling and neighbourhood satisfaction. de Jong et al. (2012) reported associations with increased physical activity and decreased obesity. This research has also indicated that people assess the relative presence of the PSDs in an environment in a similar way. Such general agreement, together with the observed cultural stability of the PSDs, suggests the possibility of a shared evolutionary basis for their development. Affordances for the eight PSDs have also been related to restoration from stress and cognitive fatigue. Serene, Sheltered, Natural, and Cohesive environments are commonly reported as the most restorative, while highly Social environments typically are the least sought-after during periods of high stress levels or cognitive fatigue (Grahn et al., 2010).

Adevi and Grahn (2012) conclude that although socio-cultural factors, such as childhood environment, deeply affect landscape preferences among adults, innate preferences due to evolutionary causes might be of equal importance. They argue that the PSDs Natural (nature in Adevi and Grahn, 2012), Diverse (rich in species), Sheltered (refuge), and Open (prospect) could indicate such evolutionary derived preferences. Similarly, Stoltz and Grahn (2021) suggest the possibility of an evolutionary basis for the PSDs based on their seemingly cross-cultural relevance and persistent reoccurrence in several empirical studies. Neither of these studies however explicitly order the PSDs along any evolutionary or developmental timeline.

## Human evolution and environmental perception

The idea that common perceptual biases and preferences might be explained from an evolutionary perspective has been investigated and discussed from various perspectives, including preferences in sexual selection (Skamel, 2003), design aesthetics (Coss, 2003), and landscape preferences (Ruso et al., 2003). Appleton (1975), through his “habitat theory,” proposed that we prefer settings inviting exploration, with signs of vital resources present. He suggested that we seek conditions offering physical protection combined with an overview of the landscape, known as the *prospect-refuge theory*. Wilson (1984) suggested the widespread *biophilia hypothesis*, the idea that we share an innate affinity and fascination for the living nature due to evolutionary causes. Orians (1986) proposed the related *savanna hypothesis*, that humans show an innate preference for the savanna biotope due to evolutionary adaptations to such conditions. Falk and Balling (2010) reported some support for this in an empirical study.

Since then, many studies have suggested links between recreation in nature and a broad range of positive health and wellbeing outcomes (e.g., Hartig et al., 2014; Egorov et al., 2016). Markevych et al. (2017) suggest that such beneficial effects work through three main pathways: (1) *mitigation* (reduction of harm, e.g., reducing exposure to air pollution or noise), (2) *restoration* (restoring capacities, e.g., attention restoration, physiological stress recovery), and (3) *instoration* (building capacities, e.g., encouraging physical activity, facilitating social cohesion). Perceived qualities, such as the PSDs, seem key to consider mainly in relation to (2) and (3), which both highlight the need for a psychological level of analysis. Focusing on restorative effects there are two main theories, both adhering to the broader idea that the way we respond to our environment has evolutionary causes. They do however explain restoration through different mechanisms, making the two approaches complementary.

The attention restoration theory (ART; Kaplan, 1995) provides a cognitively oriented approach. It focuses on our capacities for attention and distinguishes between two basic kinds, directed attention and soft fascination. It holds that our directed attention has a limited capacity that gets exhausted with overuse. Executive functions such as planning and problem solving require activation of our directed attention, as do many modern, urban environments, with plenty of sensory stimuli asking for our attention (Kaplan and Berman, 2010). Environments that instead trigger our soft fascination, such as expanded natural settings according to ART, allow our capacities for directed attention to restore (*ibid.*). The stress-reduction theory (SRT; Ulrich et al., 1991) on the other hand, presents a primarily affect-oriented approach. It emphasises the importance of autonomic responses to environmental perceptions. Evolutionary favourable settings are suggested to induce parasympathetic, i.e., stress-reducing, responses whereas adverse conditions trigger sympathetic responses, i.e., induce stress. Here, savanna-like environments are suggested to provide optimal conditions and have been linked with stress reduction when compared with urban settings (*ibid.*).

However, not all findings support the savanna hypothesis (Ruso et al., 2003; Hartmann and Apaolaza-Ibáñez, 2010). Partly this might be explained by the importance of cultural influence and childhood landscape exposure (Adevi and Grahn, 2012). We also did not emerge as anatomically modern humans, *Homo sapiens*, on the African savanna out of nowhere. Instead, we had already gone through several major phases of adaptation to changing environmental conditions. Each such phase favouring unique adaptations of our psychophysiology, thus shaping the ways in which we perceive, evaluate, and respond to our surroundings. Since then, we have developed culturally and have managed and transformed our environment on an unprecedented scale, actively reshaping our habitats. So much so that the most recent period in earth’s geological history commonly is referred to as *Anthropocene*, the epoch of humans. Thus, both the time before and after our suggested emergence on the African savanna has deeply shaped the way we perceive, make sense of, and interact with the world. Additionally, research on rehabilitation from chronic stress and cognitive fatigue suggests that long-term restoration generally go through different stages, each supported by different perceived qualities in the environment (Grahn et al., 2010). This process is often illustrated as a pyramid, in which each step build on the one below and is marked by a regained interest for outward-directed attention and social involvement (*ibid.*). Could this process somehow reflect our common developmental path?

Genome studies suggest that modern humans were cut off from an archaic ancestor some 260,000–350,000 years ago, most likely in Africa (e.g., Richter et al., 2017; Schlebusch et al., 2017). Despite great efforts to reconstruct our evolutionary origins however, the exact ancestral lineage of anatomically modern humans *(H. sapiens)* remains largely unknown (Fuss et al., 2017). Even so, there seems to be some general agreement over our overall evolutionary path (*cf.*, e.g., Wood, 1996), including the development from earlier (1) *vertebrates* into (2) *mammals* and further into tree-living (3) *primates*. Eventually, we came down from the trees and evolved into a (4) *forest-dwelling ape*. From there, we gradually started to explore the (5) *forest edge zones* and eventually completely left the forests to become (6) hunter-gatherers in a more open *savanna* landscape in which we, according to the arguably most widespread view, evolved into anatomically modern humans. Since then, we have also developed (7) *agriculture* and permanent settlements, dramatically altering our surroundings on a grand scale. Even more recently we have seen a still on-going, massive (8) *urbanisation* and a development of our social structures into increasingly larger super-structures, once again dramatically changing our habitat. Here it is proposed that these fundamental stages of human development are reflected in the eight PSDs, and in their relative restorative potential.

## Integrated habitats: An evolutionary perspective on the perceived sensory dimensions

The following is an outline in chronological order linking the eight PSDs to key transitional phases during human evolution and development as a species. This supposedly affect how they, as perceived qualities afforded in the environment, can support different stages of restoration from stress and cognitive fatigue, as well as provide a sense of meaning and wellbeing in a broader sense.

### Serenity and Shelter: Homeostasis and basic physical safety

Some properties of our neurophysiology are shared with all vertebrates and stem back several million years. As mammals we share other more distinct properties that shape our basic needs and phenomenology, and thus our environmental preferences. To stay alive, we require environmental support for basic functions such as temperature regulation, oxygen levels, the absence of threatening stimuli, as well as adequate physical shelter and protection. The autonomic nervous system could be placed at the core of the hierarchical structure of the human nervous system, suggesting an ancient origin (Wang, 2012). It handles functions such as homeostasis, blood pressure, heart rate, and respiration, that can be traced back at least to the very first mammals, if not longer (*ibid.*). They are thus extremely old, fundamental for our existence as biological beings, and necessary for the development of all our “higher” capacities. Hypothetically, the oldest and most fundamental environmental preference among the PSDs would then correspond to the Serene quality, emphasising calm and safe environmental conditions, supporting parasympathetic responses. When basic homeostatic function is established and sensory perception is directed outwards, the need for physical safety would arguably be the next immediate need realised in relation to the outer environment, reflected through the Sheltered quality in the PSD model (Figure 1).

### A Natural quality: Evolving as primates in the trees

**Figure 2:**
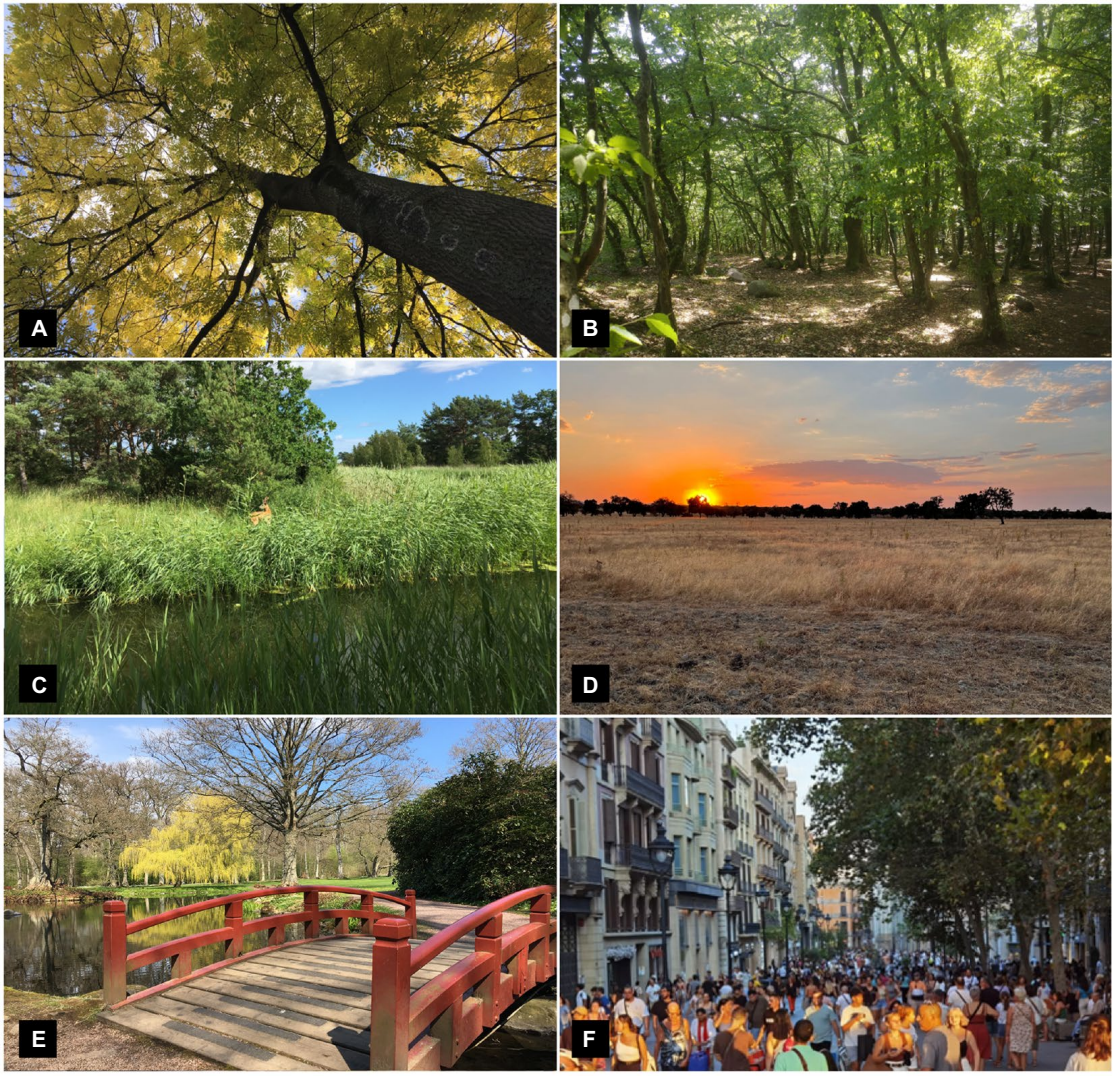
Examples of PSDs and their possible developmental roots. **(A)** Fundamental aspects of how we perceive and interact with the Natural world can be traced to adaptations to an arboreal life. **(B)** A human affinity for extended Cohesive settings could stem from adaptations to a life on the forest floor. **(C)** Adaptations to structural and biological diversity in forest edge zones might form the evolutionary basis for the Diverse PSD. **(D)** The Open PSD, with expanded vistas and plenty of room to roam, could reflect adaptions to a hunter-gatherer lifestyle in increasingly open settings. **(E)** The Cultural PSD, characterised by the distinct traces of human creative efforts, primarily reflects cultural evolution and adaptations to increasingly human-altered environments. **(F)** Although our highly social nature might reflect ancient primate traits, it is through the relatively recent urbanisation that Social affordances have increased to a previously unprecedented degree. (Photos by the author).

Several key aspects of how we perceive and relate to the characteristic properties, patterns, and features in the natural world, attributes associated with the Natural PSD (Stoltz and Grahn, 2021), can be traced back to a phase when our ancestors evolved as tree climbing primates in dense forests. Whereas the oldest known fossils of the Primate order (to which humans belong) date from around 54–55 Ma (million years ago), molecular data analyses suggest an even earlier divergence from other mammals, some 90 Ma (Tavaré et al., 2002). Regardless of exactly when, primates seem to have evolved in the trees of tropical forests (Franzen et al., 2009) and as humans we still exhibit many characteristics of adaptations to a life in trees (Figure 2A). Besides often having unusually large brains in comparison to body size (Boddy et al., 2012), primates also show an increased reliance on stereoscopic vision rather than smell and are unique among mammals in possessing trichromatic colour vision (Regan et al., 2001; Carvalho Livia et al., 2017). Consequently, many of nature’s characteristic features and patterns, such as its distinct colours or fractal patterns (Mandelbrot, 1983; Hägerhäll et al., 2004), are perceived by us largely through our highly developed sense of vision. Apart from needs to detect ripe fruits among foliage and judging distances for climbing, our visual capacities can also be linked with an acute need among our tree living primate ancestors to detect dangerous snakes in their habitat, the so-called snake detection theory (Isbell, 2006; Soares et al., 2014; Kawai and He, 2016). In addition, much of our uniquely developed hands, also of key importance in how we typically relate to and interact with our environment, can be traced back to this early phase of our evolution (e.g., Rolian et al., 2010). Some evidence also suggest that it was already in the trees, rather than on the savanna, that our bipedalism first developed (Crompton et al., 2010).

The tree-dwelling Pierolapithecus catalaunicus, that lived about 13 Ma, has been suggested as a possible last common ancestor to humans and the great apes (the Hominidae family), including our closest living relatives, the chimpanzees (Moya-Sola et al., 2004). It had specialized adaptations for tree climbing, such a wide, flat ribcage, flexible wrists, and a stiff lower spine, still present in modern humans (*ibid.*). More recent molecular evidence suggests that the Hominini tribe, which includes humans and their now extinct relatives, diverged from the Panini tribe, with the chimpanzees and bonobos, as recently as around 5–8 Ma (Crompton et al., 2010). Whenever exactly this split took place, we know that our tree-dwelling ancestors eventually left the canopies to adapt to a life on the ground. However, all such subsequent adaptations were made from the basis of our ancestor’s unique adaptations to an arboreal lifestyle, still fundamentally shaping how we perceive and interact with the world today. With such deep evolutionary roots, it might come as no surprise that perceptions of trees in particular have been linked with increased landscape preference as well as stress reduction in modern humans (Ulrich, 1986).

### A Cohesive quality: Life at the forest floor

The Cohesive PSD describes an environment perceived as a spacious, cohesive whole; an uninterrupted, expanded space, possible to explore and wander around within (Stoltz and Grahn, 2021; Figure 2B). It appears as very similar to the extent quality suggested by ART as essential to promote cognitive restoration (Kaplan, 1995; Pasini et al., 2014). Both include aspects of environmental coherence as well as scope. This PSD has been interpreted both as “a sense of forest” (Carlén et al., 2009) and as a sense of “space” (Grahn and Stigsdotter, 2010). An affinity for such a perceived quality in the environment might stem from a time when our ancestors came down from the trees but continued to dwell inside large, spacious forests, surrounded by a relatively homogenous vegetational structure. An ancestor at such an evolutionary stage could possibly be found among the various sub-species of the *Australopithecus* genus, emerging around 4 Ma, such as the *A. afarensis*. They supposedly appear relatively early on the lineage leading to anatomically modern humans, prior to a great increase in brain size (Boddy et al., 2012). Another species of the *Australopithecus* genus that has been suggested as a possible human ancestor is the *A. sediba* (Berger et al., 2010). A unique construction of its ankle and foot suggests a life primarily on the forest floor but with some traces of arboreality still present (Zipfel et al., 2011). It is suggested to have dwelled inside large forests, surviving on a mainly vegetarian diet (Henry et al., 2012).

### A Diverse quality: Foraging in patchwork landscapes

The Diverse PSD is associated with environments with varied structural features and a large diversity of plant and animal species (Stoltz and Grahn, 2021; Figure 2C). Water features and a multi-layered vegetation, varied in structure and species composition, with different habitat conditions supporting a varied wildlife can all contribute to this perceived quality (*ibid.*). An appreciation for such Diverse settings might stem from a phase of our evolution during which changing environmental conditions forced our ancestors to leave the deep forests to search for food in more mixed conditions, close to forest edges. The emergence of the Homo genus around 2 Ma is marked by a relatively sudden increase in length and body mass, with slimmer bodies allowed through a dietary shift towards more meat (Ruff et al., 1997; Ruff, 2002). These anatomical changes coincide with a major ecological change towards a more arid climate and more open landscapes, and roughly from this point on a fully terrestrial (non-arboreal) lifestyle is suggested for our ancestors (*ibid.*). A dramatic increase in brain size, possibly accompanied by the emergence of deliberate use of fire for cooking is also noted during this period (Tattersall and Schwartz, 2009; Chazan, 2017). *Homo erectus* is commonly considered a probable human ancestor emerging during this phase (e.g., Coqueugniot et al., 2004). Magill et al. (2016) studied biomarkers from a 1.8-million-year-old habitat of early *H. erectus*, concluding the presence of *“aquatic plants and protective woods in a patchwork landscape.”* The setting included a spring-fed wetland close to a forested area, all surrounded by open grassland. Animal bones with cut-marks were found “*within meters of wetland vegetation delineated by biomarkers for ferns and sedges … amid the refuge of an isolated forest patch and near freshwater with diverse edible resources.”* (*ibid.*). Possibly, it is among members of *H. erectus* that symbolic language started to develop as a response to increased needs to coordinate hunts and to distinguish between diverse environmental features, facilitated by the increased cognitive capacities allowed by the observed dietary changes (see Everett, 2017).

### An Open quality: Hunters and gatherers of open plains

The PSD Open (Stoltz and Grahn, 2021; Figure 2D) describes large open environments with unbroken vistas, few obstacles, and plenty of room to move freely. It is a popular notion that the name of the Serengeti region in northern Tanzania, home of one of the world’s most well-known wilderness reserves with prime examples of the African savanna biotope, comes from a Masai root referring to a land of “endless plains.” True or not, this might sum up the essence of this perceived quality quite well. It seems reasonable that an affinity for such Open conditions developed during a phase in which our survival increasingly depended on our skills as hunter-gatherers in increasingly open settings. In contrast to, e.g., tree living primates or forest dwelling apes, we do not only tolerate but also appreciate access to expanded and open landscapes, allowing us to roam freely and gaze far into the distance. Such environments could offer new opportunities for food provision, but also imply exposure to danger through a relative absence of shelters and hideaways. Presumably, appreciation of such conditions was widespread at the time of emergence of anatomically modern humans, *H. sapiens*, on the African savanna (Richter et al., 2017; Schlebusch et al., 2017). It is usually suggested that *H. sapiens* evolved from some of the sub-species or descendants of *H. erectus*, while brain comparisons between the two species suggest major cognitive differences (Coqueugniot et al., 2004). Although the exact lineage, period, and place of emergence still is obscured, Hublin et al. (2017) present fossil findings from Morocco, dated to be from around 315,000 years ago and interpreted as of early *H. sapiens*, with key features of modern human morphology established.

### A Cultural quality: Sedentism and agriculture

The Cultural PSD describes a managed and cultivated quality in the environment, the distinct traces of human creative effort, by past and present generations (Stoltz and Grahn, 2021; Figure 2E). The emergence of this PSD as a common environmental preference fits well with the transition from a primarily nomadic lifestyle as hunter-gatherers towards increased sedentism, and with the emergence of agriculture. Arguably, well past the establishment of modern *H. sapiens*, our habitat had been an essentially natural, i.e., uncultivated environment, with no or very few permanent traces of human activity. With the gradual introduction of sedentism and agriculture however, our typical habitat transformed into an essentially cultivated world, a world predominantly shaped and affected by human creativity and activity. Sedentism is often seen as an essential step in the evolution of more complex societies and is related to the occurrence of more permanent human traces in the landscape, such as, e.g., buildings, as well as with the development of agriculture. Snir et al. (2015) investigated a 23,000-year-old hunter-gatherer sedentary camp on the shore of the Sea of Galilee, Israel, and encountered trial cultivations indicating one of the earliest documented attempts to cultivate wild cereals. Around 12,000 years ago, most human societies employed varying degrees of ecologically transformative land use practices, *“including burning, hunting, species propagation, domestication, cultivation, and others”* (Ellis et al., 2021). Already at this time, nearly three quarters of earth’s land was inhabited and to some degree shaped by humans (*ibid.*).

### A Social quality: Urbanity and life in cities

As most primates, we represent a fundamentally social species, a trait that probably reaches way back in our evolutionary past. Our relations and interactions with others are of paramount importance for our general health and wellbeing. The Social PSD is particularly strong in bustling environments with lots of people and opportunities for social interactions and activities (Stoltz and Grahn, 2021; Figure 2F). It is often associated with what could be considered typically urban attributes, such as the presence of entertainment, restaurants, cafés, shops, etc. (*ibid*). Although for the most part of our evolution group sizes were limited, the number and diversity of Social affordances have increased dramatically through the still ongoing massive urbanisation, and even more recent digitalisation. This while affordances for Serene, Natural, and Cohesive conditions have decreased, particularly for urban dwellers. It has been suggested that we are adapted to handle group sizes up to a couple of hundred individuals, due to cognitive constraints (Dunbar, 1993). Early examples of larger urban areas include Uruk in Iraq, estimated to have had at least 40,000 inhabitants some 5,000 years ago (Nissen, 2003). First city in history reported to have passed 1 million inhabitants was ancient Rome during the centuries around year 0 (Hanson and Ortman, 2017). This was not repeated until London, England, achieved the same in 1810, followed by New York, United States, in 1875. Since then, global urbanization has been massive. More than half of the human population now resides in urban settings (UN, United Nations, Department of Economic and Social Affairs, Population Division, 2019), making present-day *Homo sapiens* a predominately urban species, a “*Homo urbanus*.” Arguably, this development is not only driven by socioeconomic necessity and population growth, but also by the fact that we, as a species, seem to value many aspects of to the kind of hyper-cultivated and hyper-social conditions characterising modern urban living environments.

## Layered needs: Implications for the relative restorative influence of the PSDs

**Figure 3:**
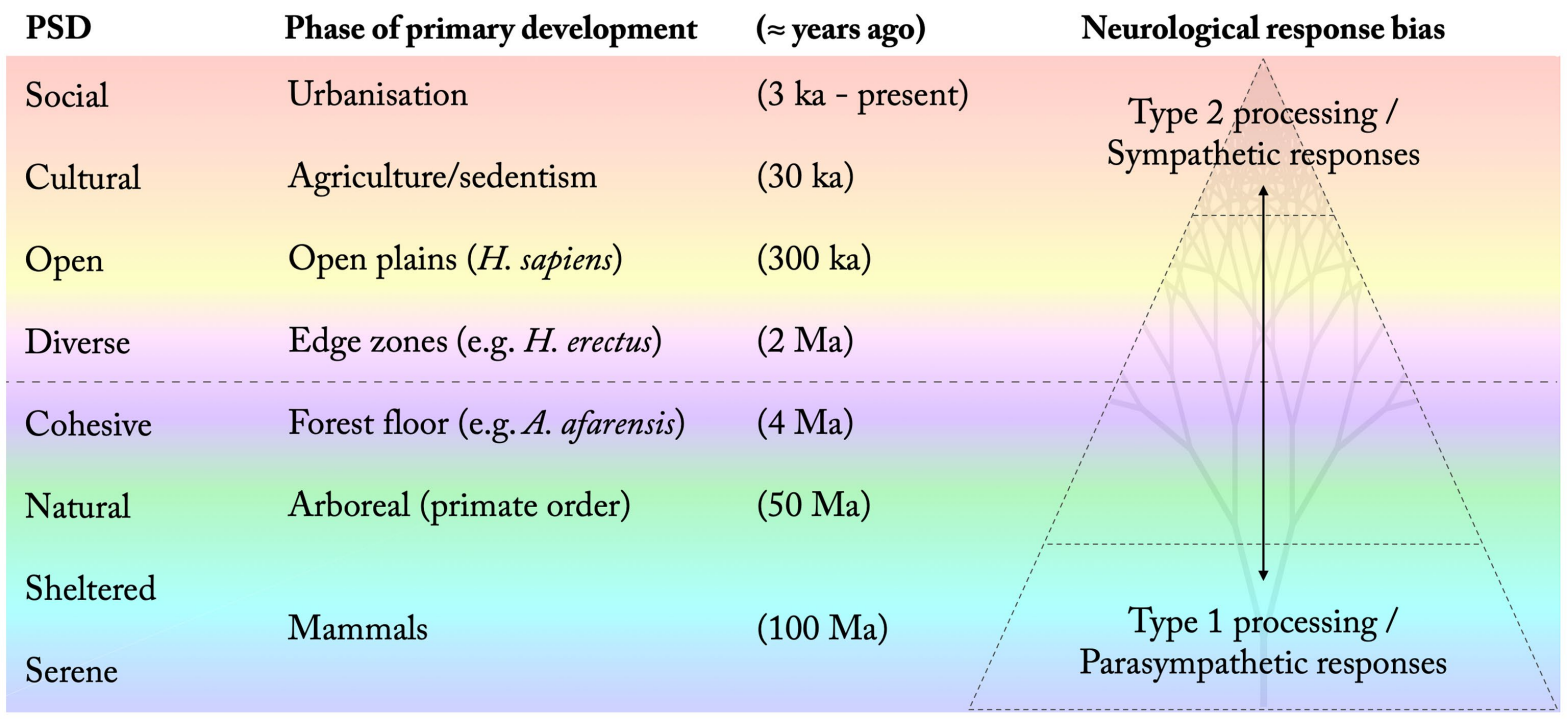
Suggested order (bottom to top) and phase associated with the development of each perceived sensory dimension (PSD). The increased complexity of our environmental relations over time is reflected in a shifted bias towards Type 2 cognition and sympathetic responses. (Time indications are estimates based on suggestions in the literature).

Figure 3 summarises the proposed order and phase of most prominent development of each PSD, both as a quality commonly afforded in our living environment and as a general preference in the population. This is related to differences in the type of neurological processes that tend to be promoted by each PSD, where more ancient qualities correspond to more autonomous functions while more recently developed preferences are associated with higher levels of cognition and increased voluntary control. This supposedly affects the relative restorative potential of each PSD (Figure 3).

Dual-processing theories of cognition suggest that autonomous, Type 1, processes provide fast default responses unless intervened by higher order, Type 2, cognition (Evans and Stanovich, 2013). Type 2 processes support abstract thinking and planning that put a heavier load on directed attention and working memory capacities, thus requirings more effort (*ibid.*). It is associated with later developed brain structures, primarily within the prefrontal cortex, while Type 1 processes are associated with structures of more ancient origin (e.g., Kahneman, 2011; Evans and Stanovich, 2013). Varga and Hamburger (2014) stresses that rather than reflecting an either-or dichotomy, Type 1 (fast, intuitive, autonomous, effortless) and Type 2 (slow, reflective, deliberate, effortful) processes exist along a gradient. Here, observed differences in relative restorative potential between the PSDs are interpreted as a gradually shifting bias in the type of neurological processes promoted by each subsequently developed quality (Figure 3). This model for nature-based restoration appears in overall agreement with the basic proposition by ART of two different kinds of attention, where extended natural environments allow our capacities for directed attention to restore, whereas less natural, especially urban, environments tend to exhaust these capacities (Kaplan, 1995; Kaplan and Berman, 2010). ART’s concept of “soft fascination” (*ibid.*) would then correspond to environments favouring Type 1 processes while environments and activities demanding a lot of “directed attention” would be associated with a higher bias towards Type 2 processes. It also agrees with the assertion by SRT (Ulrich et al., 1991) that evolutionary favourable, safe, and natural settings - presumably corresponding to PSDs of more ancient origin - tend to trigger parasympathetic autonomic responses, whereas novel conditions, in particular urban environments, more often generate sympathetic responses, i.e., induce stress.

In overall agreement with both ART and SRT, the proposed model present an integrative approach that potentially could be used to analyse and understand environments’ relative restorative influence from both an affective and a cognitive perspective within a unified evolutionary framework. Through the hierarchical ordering of the PSDs it also suggests how different perceived qualities can aid different stages of restoration. The restorative process has been proposed as passing through distinct phases while moving towards a regained interest for outward-directed attention and social involvement, and hence an increased tolerance and appreciation for the Social PSD (Grahn et al., 2010). Several studies on the PSDs in relation to this process have been made. This research show that people in strong need of restoration primarily seek out environments perceived as Serene, Sheltered, and Natural (e.g., Grahn et al., 2010; Memari et al., 2017; Stigsdotter et al., 2017; Pálsdóttir et al., 2018). Serene and Sheltered conditions seem particularly important to support early stages of rehabilitation while the Social quality usually is considered the least restorative and most demanding of the PSDs in the mentioned studies. This is all in agreement with the proposed model, that places Serene as a fundamental PSD for restoration, together with Sheltered and Natural. This is suggested to be followed by a growing demand for the Cohesive PSD, that would indicate a phase of increased mobility and exploration of the environment, after the fulfilment of more fundamental restorative needs. This as well seems to agree with empirical findings (see e.g., Grahn et al., 2010).

**Figure 4:**
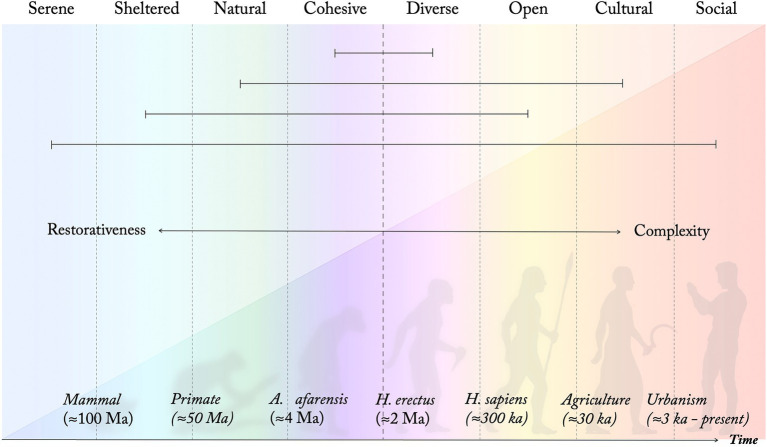
Eight perceived sensory dimensions of different development and restorative influence. As present-day perceived qualities and design principles they are understood along four axes of opposing qualities, connected with horizontal lines following the PSD model (Figure 1). (Schematic illustration. Time and species indications are estimations based on suggestions in the literature).

As the restoration/rehabilitation process proceeds, Diverse, Open, Cultural, and Social environments gradually become more important (*ibid.*). While some studies (e.g., Ottosson, 2001; Memari et al., 2017) suggest a negative influence of the Diverse PSD on restoration, others suggest that it plays an important role (Grahn and Stigsdotter, 2010; Stigsdotter et al., 2017). The period around 2 Ma, where the Diverse PSD is suggested to emerge through adaptations to a diverse patchwork landscape, marks a shift in the model from the four more restorative PSDs to what perhaps could be considered the four more stimulating, but also cognitively more challenging, qualities (marked by a dashed line in Figures 3, 4). As described, this is a period associated with a dramatic increase in brain size of our ancestors, presumably a result of adaptations to a significantly more complex and diverse habitat. This is also a period that has been associated with the first developoment of symbolical language (Everett, 2017). The suggested model indicates that the relative difference in terms of cognitive demand and restorative influence between a Cohesive and relatively uniform space and a more Diverse setting, although significant, still is relatively low compared to that between a Serene and a highly Social environment. It also suggests that highly Diverse settings might be more beneficial after the most severe stages of stress and mental exhaustion have passed, which seems supported by empirical findings (Grahn et al., 2010). The proposed model could thus explain the somewhat contradictory reports regarding the Diverse PSD in relation to restoration in empirical studies, and highlights that rather than a quality being restorative or not, different perceived qualities might support different stages of restoration.

While the Sheltered PSD often is found to be of particular importance to support restoration, the Open PSD is associated with potential exposure of the individual to the eyes of others (humans or animals), and thus to potential threats (Stoltz and Grahn, 2021). Perhaps this is part of the reason why this PSD has not been strongly associated with restoration in the mentioned studies. However, it is possible that the Open quality in direct proximity to Shelter, effectively supporting a prospect-refuge relationship as described by Appleton (1975), can function as an efficient combination to support restoration, which is suggested in some studies (see e.g., Sonntag-Öström et al., 2015; Stigsdotter et al., 2017; Pálsdóttir et al., 2018). An Open quality is suggested as the PSD following on the Diverse quality in the proposed restoration gradient (Figures 3, 4). If the origin of the Diverse PSD is linked to mixed settings with a relatively dense compilation of forested areas, open grasslands, and water features (*cf.* e.g., Magill et al., 2016), this would typically describe settings affording plenty of Shelter as well. Interestingly, when looking at the relations between the PSDs as design principles suggested in the PSD model (Figures 1, 4), Diversity and Shelter represent adjacent qualities, supposedly closely related and synergetic when combined (*ibid.*). The Appendix further develops some environmental planning and design heuristics based on such a synthesis of the proposed evolutionary perspective and the PSD model.

Although some studies (e.g., Xu et al., 2018) suggest that certain cultivated landscapes might offer support for restoration, the Cultural PSD is typically not strongly associated with restorative effects in the mentioned empirical studies. However, it often seems to grow in importance during later stages of the restoration process (Grahn et al., 2010). Even though the suggested time periods in the proposed model (Figures 3, 4) are rough estimations, it is interesting to note that the duration of each proposed developmental phase seems to diminish exponentially. Counting the last 4 million years of evolution, we have spent more than 99% of the time in what could be considered fundamentally natural environments, i.e., environments mainly unaltered by humans. Adaptations to such conditions thus define most of our genome, creating a convincing foundation for evolutionary grounded theories for nature-based restoration. The Cultural PSD seems to imply a shift in that it is a phase primarily defined by us changing and adapting our environment, rather than the other way around. Together with the massive development of the Social PSD through the even more recent urbanisation, it is distinguished from previous stages in implying relatively minor changes of our genome, due to the relatively short timescale. These two PSDs, for now, thus primarily reflect cultural, rather than genetic, evolution and adaptation. And although often rewarding and stimulating, urban environments are typically associated with increased stress levels and cognitive fatigue when compared to natural surroundings (Ulrich et al., 1991; Kaplan and Berman, 2010). Similarly, the Social PSD is usually rated as the least restorative and most demanding of the PSDs in empirical studies (e.g., Grahn and Stigsdotter, 2010; Stigsdotter et al., 2017; Pálsdóttir et al., 2018). This might in part reflect how increased complexities in our social relations, not least related to group size, put increased pressure on our neurological capacities (cf. Dunbar, 1993).

Arguably, it is primarily through the relatively recent development of the Cultural and Social PSDs that the previous evolutionary phases in a sense are transcended and the PSDs take on their present form as perceived environmental “colours” (Stoltz, 2019; Stoltz and Grahn, 2021), or as more abstract design principles, rather than being understood as specific biotopes or narrowly defined habitat conditions. In an increasingly cultivated world, the separation of the Natural from the Cultural has become increasingly distinct; a process with deep effects on human meaning-making. The more recent urbanisation (and even more recent digitalisation) has further highlighted the need for peaceful, Serene environments, free from disturbances and with few or no Social affordances. This distinction is reflected in the opposite relations suggested between the Social and the Serene qualities in the PSD model (Figures 1, 4). This development has arguably further increased the perceived gap between us and our natural roots with important consequences both for our individual mental and physical wellbeing and for the biosphere at large, considering how this impacts our individual and collective behaviours. However, as Natural and Serene affordances have decreased, the value of these perceived qualities in the environment has become increasingly clear, evident not least through the large body of research surrounding the influence of such settings on our health and wellbeing developed during the past decades.

## Conclusions

Our ancestors’ multiple adaptations to novel conditions, and the order of these transitions, shape who we are and how we perceive and interact with the world. This is reflected in eight perceived sensory dimensions (PSDs), suggested by previous research to describe key sensory needs in recreational outdoor environments. The proposed model includes transitions taking place both before and after our suggested appearance as anatomically modern humans at the African savanna. It thus integrates a “pre-savanna” phase, focused on evolution in forest settings (corresponding to the PSDs Serene, Sheltered, Natural and Cohesive), an “edge zone” or “patchwork” phase (linked with the PSD Diverse), an “open plains” or “savanna” stage (PSD Open), as well as a “post-savanna” developmental phase with permanent settlements and cultivation (PSD Cultural) and even more recent adaptations to urban living conditions (PSD Social). This developmental order suggests a gradient from more restorative (promoting of Type 1 processes and parasympathetic autonomic responses) towards more demanding (promoting of Type 2 processes and sympathetic autonomic responses) PSDs. This implies a hierarchical order where the PSDs might support different stages of restoration. The model seems to agree with empirical findings regarding human evolution and development as a species, as well as the relative importance of different perceived qualities during different stages of restoration. It also seems compatible with currently established theories explaining restorative mechanisms. It could thus be of potential use when shaping modern human habitats, green areas, health care settings, recreational forests, or other outdoor environments, to support both restorative and instorative recreational functions. Not the least this seems relevant in the light of current global trends regarding urbanisation and lifestyle-dependent illness, often related to stress and/or cognitive fatigue.

## Data availability statement

The original contributions presented in the study are included in the article/supplementary material; further inquiries can be directed to the corresponding author.

## Author contributions

JS is sole contributor of this work and has approved it for publication.

## Funding

Part of this research has been funded by the FORMAS Research Council; the project ‘Sustainable outdoor living environments—systematic interdisciplinary studies of health effects and impact on social inequalities’ (D-nr 2019-01916).

## Conflict of interest

The author declares that the research was conducted in the absence of any commercial or financial relationships that could be construed as a potential conflict of interest.

## Publisher’s note

All claims expressed in this article are solely those of the authors and do not necessarily represent those of their affiliated organizations, or those of the publisher, the editors and the reviewers. Any product that may be evaluated in this article, or claim that may be made by its manufacturer, is not guaranteed or endorsed by the publisher.

## Figures and Tables

**Figure A1:**
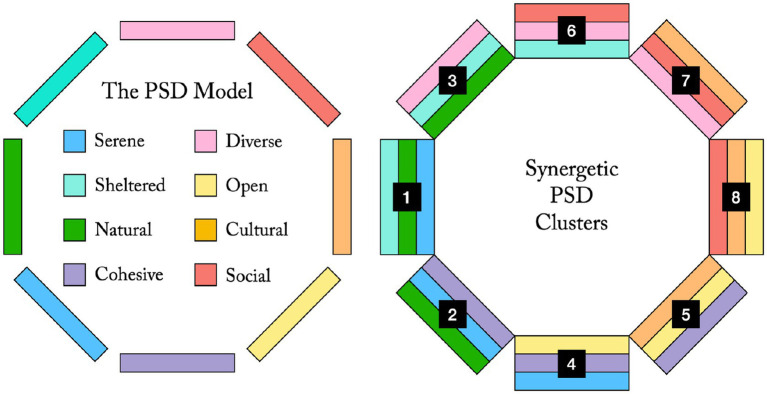
Support for three adjacent qualities in the PSD model has been suggested as a simple heuristic for strong recreational function, from a perceived qualities perspective. This gives eight synergetic combinations of three PSDs each (Clusters 1–8).

**Figure A2:**
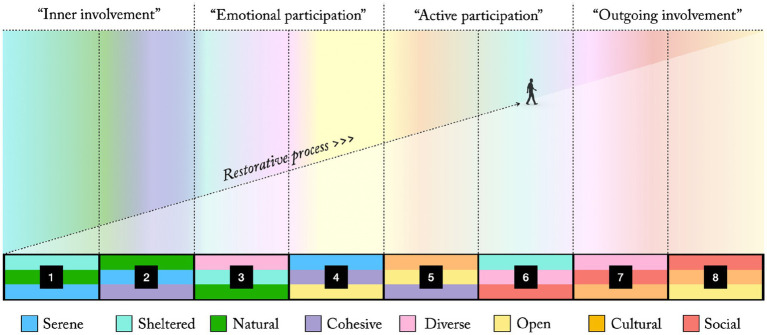
A sensory opportunity spectrum (SOS) with eight PSD combinations (Cluster 1–8) supporting different stages of restoration, roughly mapped unto four phases of the restorative process suggested by Grahn et al. (2010).

## Appendix: Possible environmental planning and design heuristics

If the *sensory opportunity spectrum (SOS)* of an environment is the possible sensory experiences afforded to visitors, this could be analysed in terms of affordances for the eight PSDs. Figure 4 could be seen as describing a simple SOS, with the eight PSDs in consecutive order following the suggested evolutionary timeline. This gradient could be interpreted spatially and taken as a simple guide for environmental planning and design, when aiming for gradual transitions between settings supporting different stages of restoration. However, the PSDs are rarely perceived in isolation. Instead, each PSD can be supported to various degrees, creating different combinations of stronger and weaker qualities (Stoltz and Grahn, 2021). It might thus be relevant for practical purposes to consider which combinations of PSDs to support in an environment. There is usually a tension between opposite qualities in the PSD model (Figure 1) while adjacent qualities often support each other and act synergistically (*ibid.*). This is the basis for the proposed heuristic that when three adjacent qualities are well-supported in an environment it offers strong aesthetic function and high recreational potential (Stoltz and Grahn, 2021; Figure A1).

If arranging these eight supposedly synergetic PSD combinations (Clusters 1–8, Figure A1) according to the strongest restorative influence of the *least* restorative quality they contain (following the proposed evolutionary model, Figures 3, 4), we get the more detailed SOS presented in Figure A2. (For the three clusters containing the Social dimension the remaining PSDs, that are not the same between clusters, are considered in the same manner until all clusters are ordered). This might illustrate which PSD combinations that could be used to support different stages of restoration. Local parts of an environment (e.g., a rehabilitation garden or similar care setting, a recreational forest, an urban park, a residential area) could then be designed with these PSD combinations in mind, roughly following the spatial layout and consecutive order suggested in Figure A2. This would theoretically offer support for smooth transitions between different levels of restoration through gradually increasing sensory challenges while ensuring effective and synergetic local combinations of perceived qualities. It could also aid to ensure aesthetic diversity in the larger environment, supporting different and complementary sensory needs. Arguably, environments providing such an overall mix of perceived qualities would have good chances to be rated as attractive habitats by many modern humans.

This synthesis of the proposed evolutionary perspective and the PSD model seems to preserve an overall fit with the suggested evolutionary path (Figure 4). The PSD combination suggested as most restorative (Cluster 1, Figure A2) describes a Serene, Natural, and Sheltered environment that would fit well for the dense forest settings of our early primate ancestors (see “A Natural quality: Evolving as primates in trees”). The following Cluster 2, still Serene and Natural, but now instead of Shelter emphasising a Cohesive quality inviting increased ground level exploration, better describes the more spacious forest habitat of, e.g., *A. afarensis* or *A. sediba* (see “A Cohesive quality: Life at the forest floor”). Cluster 3, also Natural, but now in combination with Shelter and Diversity, fit well with the diverse patchwork habitats around forest edges suggested for early *Homo* (see “A Diverse quality: Foraging in patchwork landscapes”). The following Clusters 4 and 5 (Figure A2) both describe Cohesive, Open settings. The first with an emphasis on Serenity, possibly describing a pristine open savanna with plenty of hunting and foraging opportunities for early *H. Sapiens* (see “An Open quality: Hunters and gatherers of open plains”). The latter, instead with a pronounced Cultural influence, rather evokes a pastoral setting (see “A Cultural quality: Sedentism and agriculture”). It thus seems that the proposed developmental perspective is largely compatible with the modern interpretation of the PSDs as more abstract affordance classes or design principles, expressed in the PSD model. A synthesis of these two perspectives might hence offer useful guidance in environmental evaluation, planning, and design from a broad perceived qualities perspective.
